# Semaglutide Attenuates Type 2 Diabetes-Induced Neurotoxicity by Modulating Neuroinflammation, Oxidative Stress, and Neuroapoptosis

**DOI:** 10.3390/life16081317

**Published:** 2026-08-12

**Authors:** Abdulellah Saad Alharbi, Abdulaziz Arif A. Alshammari, Mai B. Alwesmi, Vasudevan Mani

**Affiliations:** 1 Department of Pharmacology and Toxicology, College of Pharmacy, Qassim University, Buraydah 51452, Saudi Arabia; 451114194@qu.edu.sa (A.S.A.); aziz.alhmzani@gmail.com (A.A.A.A.); 2 Department of Medical-Surgical Nursing, College of Nursing, Princess Nourah bint Abdulrahman University, Riyadh 11564, Saudi Arabia; mbalwesmi@pnu.edu.sa

**Keywords:** semaglutide, diabetes mellitus, neuroinflammation, oxidative stress, neuroapoptosis

## Abstract

Type 2 diabetes mellitus (T2DM) is a widely distributed dysmetabolic condition increasingly linked to neurocognitive decline, neuroinflammation, oxidative stress, and neuronal apoptosis. Chronic hyperglycemia and insulin resistance disrupt homeostasis in the central nervous system, contributing to neurodegenerative processes. This study evaluated the effects of semaglutide (SMG), a glucagon-like peptide-1 receptor agonist, on T2DM-associated neurobehavioral and biochemical alterations using a rat model. T2DM was induced by nicotinamide–streptozotocin, followed by oral administration of SMG at a dose of 1.44 mg/kg for one month. Cognitive function was evaluated using the elevated plus maze (EPM) and novel object recognition (NOR) paradigms. Fasting blood glucose and body weight were monitored throughout the experiment. Neuroinflammatory markers (COX-2, TNF-α, and IL-6), oxidative stress biomarkers (MDA, GSH, and catalase), and apoptosis-associated proteins (Caspase-3, Bax, and Bcl-2) were measured in brain tissue homogenates using ELISA. Diabetic rats exhibited marked cognitive deficits, hyperglycemia, increased neuroinflammatory markers, and altered apoptosis-related biomarkers, with reduced Bcl-2 expression. Treatment with SMG significantly improved learning and memory performance, fasting blood glucose, and body weight. At the molecular level, SMG treatment was associated with lower levels of MDA, TNF-α, Bax, IL-6, COX-2, and Caspase-3, together with higher levels of Bcl-2, catalase, and GSH compared with untreated diabetic rats. Overall, oral SMG treatment was associated with improved cognitive performance and metabolic control, accompanied by attenuation of diabetes-associated neuroinflammatory, oxidative stress, and apoptosis-related alterations in brain tissue. However, the present study was not designed to distinguish direct neuroprotective effects from changes secondary to improved metabolic control. Further studies are required to clarify the underlying mechanisms and determine the translational relevance of these findings.

## 1. Introduction

**Figure 1:**
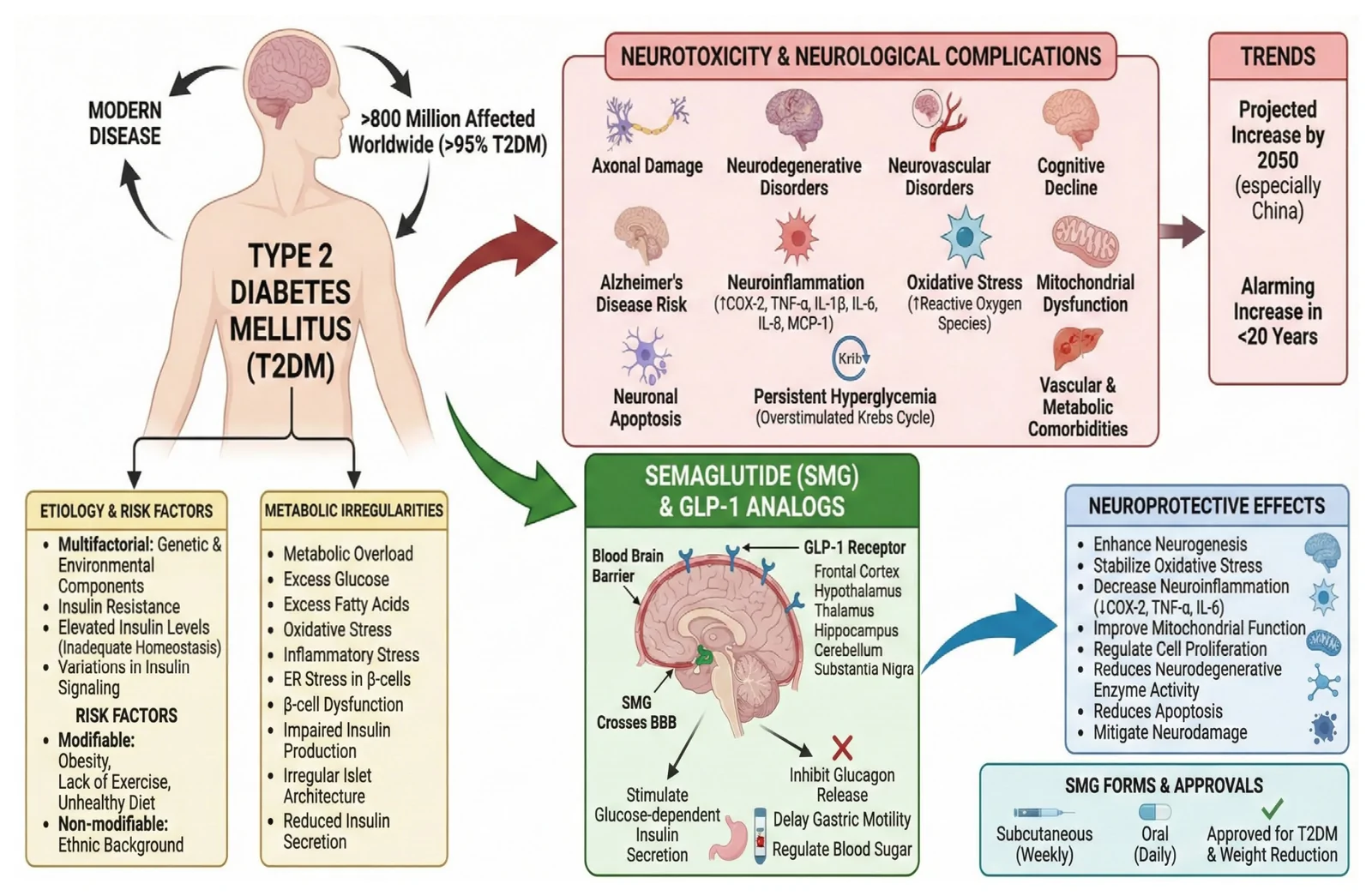
A schematic overview of how type 2 diabetes causes metabolic and neurological issues, and how SMG offers neuroprotection. T2DM-related insulin resistance, hyperglycemia, and lipid overload lead to oxidative stress, neuroinflammation, mitochondrial problems, and cognitive decline. SMG crosses the BBB, improving glucose control, reducing inflammation and oxidative stress, and providing neuroprotection.

Type 2 diabetes mellitus (T2DM) is considered to be a modern disease that occurs in modern society. According to recent estimations by the Collaborations on Risk Factors, more than 800 million people worldwide have been affected by diabetes, with more than 95% affected by T2DM [1]. T2DM is not an acute condition but is always chronic, arising from multifactorial etiologies, comprising both genetic and environmental components [2]. T2DM is always a multifaceted condition characterized by insulin resistance and elevated insulin levels, which are always inadequate to maintain proper glucose homeostasis. Furthermore, irregularities in insulin signaling, with variations in insulin levels, are thought to underlie many metabolic irregularities that occur in diabetes, with reduced efficiency in glucose utilization [3]. The trend, prevalence, incidence, mortality, and disability-adjusted life years (DALYs) with respect to T2DM are expected to record tremendous increases by 2050, especially in China. The alarming trend is observed in people under 20 years, where it has already increased by more than threefold since 1990, with further increases expected by 2050 [4]. The risk factor remains multifaceted, with an intricate interplay among environmental, metabolic, and genetic factors, resulting in an ever-increasing number of T2DM patients. Though non-modifiable risk determinants, such as ethnic background, have always been proven to have significant predispositions to this condition, it has been observed through many epidemiological studies that T2DM can always be prevented by managing major peripheral risk determinants, such as obesity, lack of regular exercise, or unhealthy dietary habits [5,6,7]. Metabolic overload, with excess glucose and excess fatty acids, always occurs, which always leads to oxidative, inflammatory, or endoplasmic reticulum stress in β-cells. β-cell dysfunction always leads to impairments in insulin production, irregularities in islet architecture, and a gradual reduction in insulin secretion. Such pathological irregularities consistently contribute to the triggering of T2DM [5] (Figure 1).

Diabetes mellitus may result in numerous complications of the nervous system, such as axonal damage, neurodegenerative and neurovascular disorders, and widespread cognitive decline. Moreover, most individuals with diabetes experience multiple vascular and metabolic comorbidities. When these conditions are combined with suboptimal glycemic control, they considerably hasten the development of neurological complications [8]. Persistent hyperglycemia additionally overstimulates the Krebs cycle, consequently generating an excessive quantity of reactive oxygen species [9]. Our previous research has demonstrated increased Tumor necrosis factor-alpha (TNF-α) and Cyclooxygenase-2 (COX-2) expression in the brain tissues of Streptozotocin (STZ)-induced diabetic rats [10]. In individuals with T2DM, monocytes and macrophages exhibit elevated concentrations of principal pro-inflammatory mediators, notably Monocyte Chemoattractant Protein-1 (MCP-1), Interleukin-6 (IL-6), Interleukin-8 (IL-8), and Interleukin-1 beta (IL-1β), among others [11]. Furthermore, mice deficient in COX demonstrate protection against neuropathic impairments associated with diabetes, including nerve conduction deficits and diminished blood flow around the myelin sheath, compared with diabetic mice with COX-2 expression. These conclusions indicate that COX-2 is intricately involved in maintaining normal nerve function [12,13]. T2DM is correlated with an elevated risk of developing cognitive–neural deterioration and Alzheimer’s disease (AD) [14]. Cognitive impairment has been documented in older and adolescent individuals with T2DM based on assessments such as the Mini-Mental Status Examination (MMSE) [15,16] (Figure 1). Furthermore, Malondialdehyde (MDA) indicates lipid peroxidation and oxidative stress, elevated in neurological disorders, signaling increased damage. Catalase, key in detoxifying hydrogen peroxide, shows reduced activity in these diseases, hinting at weakened antioxidant defense. Reduced glutathione (GSH), a vital intracellular antioxidant, varies across conditions, remaining stable or rising, possibly as a compensatory response to stress [17].

Glucagon-like peptide-1 receptor agonist (GLP-1) is a naturally occurring peptide hormone composed of 30 amino acids that plays a central role in maintaining glucose balance by enhancing glucose-triggered insulin secretion, reducing glucagon release, and suppressing gastric motility [18,19]. GLP-1 analogs can cross the blood–brain barrier (BBB) and bind to the GLP-1 receptor in several brain regions, involving the frontal cortical regions, hippocampal region, hypothalamus and thalamus regions, substantia nigra region, and cerebellum region, modulating various pathways within the Central Nervous System (CNS), such as inflammation, mitochondrial function, and proliferative activity [20,21]. Apart from their role in metabolic dysregulation, GLP-1 receptor agonists have been considered to possess significant neuroprotective effects by enhancing neurogenesis, stabilizing oxidative stress, and decreasing neuroinflammation [19]. Semaglutide (SMG) is a newer agent in this drug class and remains the only GLP-1 receptor-targeting compound approved in both subcutaneous and oral forms [22]. SMG, administered weekly, was approved in the US and Europe for the management of T2DM. Later, a daily oral formulation gained approval between 2019 and 2020. For weight reduction, a weekly dose is used, based on clinical trials showing more pronounced reductions in body weight relative to placebo and liraglutide [23,24]. In addition, in T2DM patients with high cardiovascular risk, SMG treatment significantly reduced the incidence of adverse cardiovascular events relative to placebo, thereby indicating the cardiovascular noninferiority of the intervention SMG [25]. Findings from a recent TriNetX-based cohort study indicate that the use of tirzepatide and SMG is correlated with a diminished risk of recurrent acute pancreatitis in patients with T2DM or obesity [26,27]. Furthermore, SMG has been linked to decreases in oxidative stress and improvements in mitochondrial function [20]. Antioxidants protect against neurodegeneration by limiting oxidative damage and harmful free radicals while also influencing various signaling pathways and regulating gene expression [28] (Figure 1).

SMG is available as both injectable and oral formulations. Unlike the injectable formulation, oral SMG is a clinically distinct preparation whose systemic absorption depends on the co-formulated absorption enhancer SNAC (sodium N-(8-[2-hydroxybenzoyl]amino) caprylate), resulting in pharmacokinetic characteristics that differ from those of subcutaneous administration. Although previous studies have reported beneficial cognitive or neuroprotective effects of SMG, the available evidence remains fragmented by route of administration, experimental model, target tissue, and outcome measures. Studies investigating cognitive outcomes have primarily used injectable semaglutide, whereas the study most closely matching the oral formulation and dose used in the present study evaluated spinal cord outcomes in a neuropathic pain model rather than brain-related cognitive function [19,20,21,29,30]. Moreover, the combined assessment of cognitive performance with neuroinflammatory, oxidative stress, and apoptosis-related biomarkers in brain tissue has not been adequately characterized following oral administration of clinically available semaglutide in a T2DM model. Therefore, the present study investigated the effects of an orally administered, commercially available formulation of SMG (Rybelsus^®^) on cognitive performance using the elevated plus maze (EPM) and novel object recognition (NOR) tests, together with an integrated assessment of neuroinflammatory (COX-2, TNF-α, and IL-6), oxidative stress (MDA, GSH, and catalase), and apoptosis-related (Bax, Bcl-2, and Caspase-3) biomarkers in a nicotinamide/streptozotocin-induced rat model of T2DM. Given the increasing clinical use of oral SMG in T2DM management, these findings may provide clinically relevant preclinical evidence regarding its association with diabetes-related neurobehavioral and biochemical alterations.

## 2. Materials and Methods

### 2.1. Chemicals

STZ and nicotinamide hydrochloride (NTH) were obtained from Ambeed (Arlington Heights, IL, USA), while all ELISA kits used for rat tissue analyses were supplied by MyBioSource (San Diego, CA, USA), both from the USA. In addition, SMG (Rybelsus^®^; Novo Nordisk Saudi Arabia, As Sahafah Riyadh, Saudi Arabia) was obtained from a local pharmacy chain in Saudi Arabia. SMG tablets were finely crushed and freshly suspended in normal saline immediately before oral administration.

### 2.2. Animals

**Figure 2:**
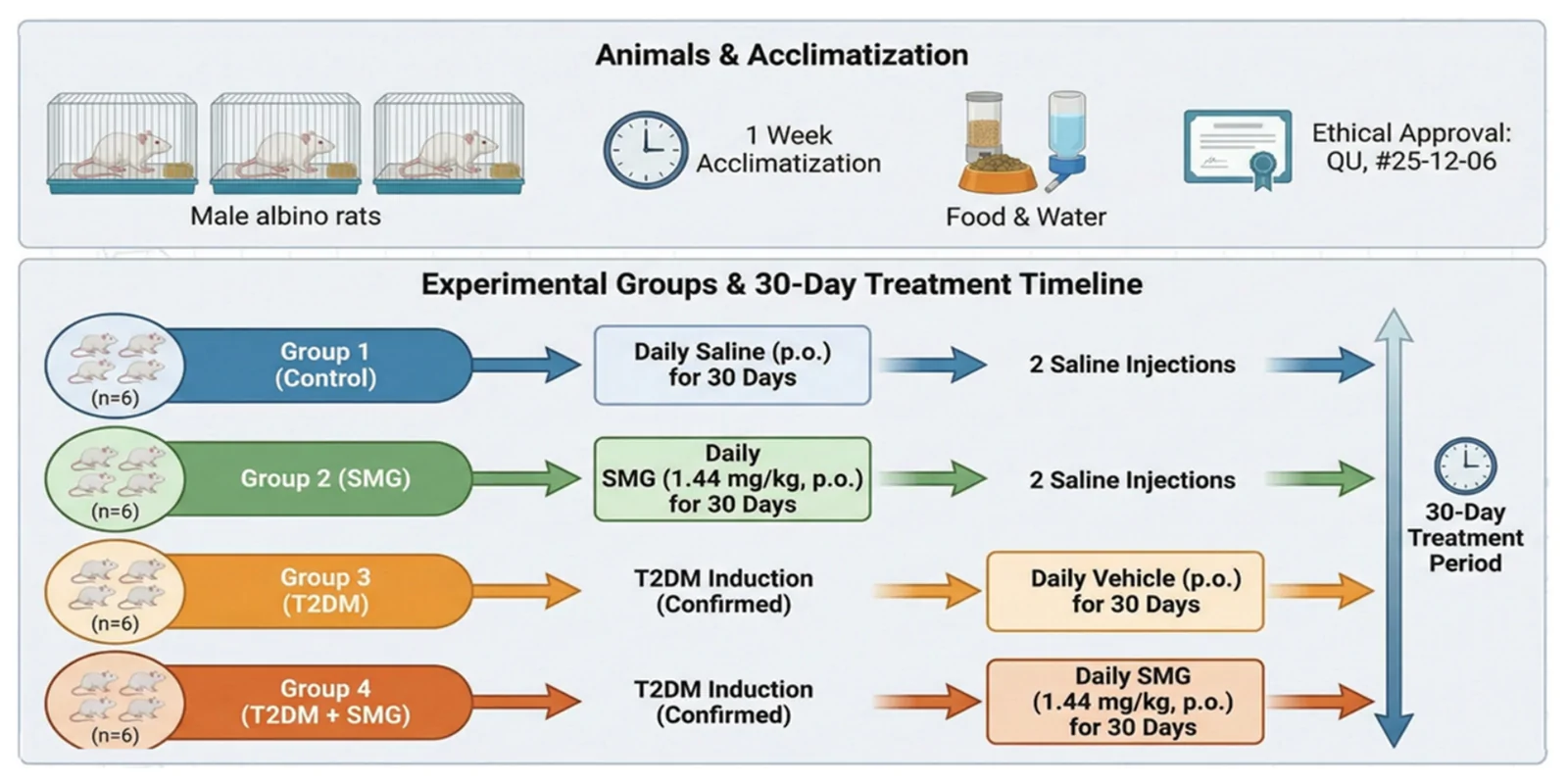
Experimental design involved 24 adult male albino rats, divided into four groups (*n* = 6): control, SMG, T2DM, and T2DM + SMG. T2DM was induced via nicotinamide–streptozotocin, with diabetes confirmed before treatment. SMG (1.44 mg/kg, p.o.) or vehicle was given daily for 30 days. The timeline shows induction, confirmation, and treatment phases.

A total of twenty-four adult male Sprague–Dawley albino rats were obtained from the animal research house in the Pharmacology and Toxicology department laboratories at Qassim University (QU). The animals, aged 11–12 weeks and weighing 170–200 g, were allocated in a random manner into four experimental groups, each containing six rats. The rats were kept three per cage in a regulated laboratory environment with a 12 h light/12 h dark schedule and continuous access to feed and water. Prior to treatment, the rats were allowed to acclimate to the laboratory environment for 1 week. All animal usage and experimental techniques were permitted by the QU (Approval No. 25-12-06), as shown in Figure 2.

The number of animals allocated to each experimental group (*n* = 6) was established before the study, based on recommendations from the Institutional Animal Ethics Committee, supported by statistical power calculations and evidence from previous studies. This approach ensured sufficient statistical power to identify meaningful treatment effects while adhering to the principles of Replacement, Reduction, and Refinement (3Rs) by avoiding unnecessary animal use. To enhance the rigor, transparency, and reproducibility of the study, all experimental procedures were designed and reported in accordance with the ARRIVE 2.0 guidelines, with particular emphasis on compliance with the ARRIVE Essential 10 recommendations [31].

### 2.3. T2DM Induction

Prior to induction, all rodents were maintained under a 12 h fasting condition. NTH was dissolved in normal saline, whereas STZ was freshly prepared in cold citrate buffer (0.1 M, pH 4.5) immediately before administration. T2DM was established via intraperitoneal injection of nicotinamide, followed fifteen minutes later by STZ at specified dose levels of 120 mg/kg and 60 mg/kg in the same order. This sequential procedure reliably induces diabetes. Hyperglycemia was confirmed seventy-two hours post-injection by assessing fasting blood glucose concentrations, which were measured by glucometer (Accu-Chek, Roche, Germany). Rats with glucose concentrations ≥127 mg/dL were classified as diabetic [10].

### 2.4. Study Design and Treatment Schedule

The Albino rats were allocated in a random manner into four groups (*n* = 6 each):

Group 1 (Control): Administered normal saline (vehicle) p.o. for 30 days and two injections of saline, similar to the T2DM induction protocol.

Group 2 (SMG): Received SMG (1.44 mg/kg, p.o.) daily for 30 days and two saline injections, like the T2DM group.

Group 3 (T2DM): The T2DM rat group, following confirmed induction of diabetes, was administered the vehicle orally for a duration of 30 days.

Group 4 (T2DM + SMG)**:** Induced with T2DM as above and treated with SMG (1.44 mg/kg, p.o.) daily for a month (Figure 2).

The SMG dose (1.44 mg/kg, p.o.) was selected based on a previously published rat study that demonstrated efficacy and safety at this dose in a diabetic model [29]. Weekly measurements of body weight and fasting blood glucose were obtained during the treatment period.

To minimize potential experimental bias, investigators conducting the behavioral assessments and biochemical analyses were blinded to the treatment allocation. Group identities were revealed only after completion of data collection and analysis.

### 2.5. Neurobehavioral Assessments

#### 2.5.1. Elevated Plus Maze (EPM)

To evaluate the cognitive function of the rats, the EPM test was performed, with performance quantified by transfer latency (TL) [32]. It consists of four arms, two unprotected (the ends considered as a starting point and oriented away from the central platform) and the remaining enclosed (40 cm high walls), shaped like a plus sign (+) (each 50 × 10 cm). The maze is elevated 50 cm above the ground and made of wood. The test was conducted in two phases. Training phase (Day 27): Each participant was permitted to freely explore the maze for a duration of 5 min. Testing phase (Day 28): The same rat was reintroduced at the same starting point, and the session was recorded. The TL, the time it takes for the rat to enter a closed arm with all four paws, was measured as an indicator of memory performance. The maze was wiped with alcohol after each rat in all phases [33].

#### 2.5.2. Novel Object Recognition (NOR)

The NOR paradigm was used to assess recognition memory [34]. It consists of three phases conducted on Days 29 and 30. Habituation (Day 29): every single rat was placed individually in an empty arena (without objects) and allowed to explore it to acclimate to the environment. Training (Day 30—Morning): Rats were placed in the same arena containing two similar objects and were allowed to explore them. Testing (Day 30—3 h later): A single familiar object was substituted with a novel object. Each phase of these three phases took 5 min. The exploratory duration of the novel object and the discrimination index were recorded as indicators of memory performance. The arena was wiped with alcohol after each rat in all phases. The percentage discrimination index (PDI) was calculated as follows: PDI = (ENO − EFO)/(ENO + EFO) × 100, where ENO represents the exploration time of the novel object, and EFO represents the exploration time of the familiar object. The arena was wiped with alcohol after each rat during all phases of the test [33].

### 2.6. Blood Glucose Analysis

Blood glucose levels were evaluated using a standard glucose assay. A sterile needle was utilized to puncture the tail vein for accurate blood collection, and glucose measurements were recorded using a Roche Accu-Chek glucometer in accordance with the manufacturer’s instructions. Blood glucose levels were measured weekly throughout the treatment period. Rodents demonstrating fasting glucose levels exceeding 126 mg/dL were classified as being T2DM diabetic [10].

### 2.7. Biomolecular Evaluations

#### 2.7.1. Tissue Preparation

Upon completion of the NOR assessment, animals were anesthetized (ketamine 100 mg/kg and xylazine 10 mg/kg, i.p.) and euthanized by cervical dislocation. The brains were rapidly excised, rinsed, and homogenized in ice-cold phosphate-buffered saline (PBS; pH 7.4, maintained at 4 °C) using a mechanical tissue homogenizer. The homogenized samples were subsequently centrifuged at 4000 rpm for 10 min, and the clear supernatants were carefully collected and preserved at −80 °C for subsequent analyses. Protein levels in the specimens were determined by the Biuret colorimetric method using a commercial kit (Crescent Diagnostics, Jeddah, Saudi Arabia).

#### 2.7.2. Bioassay of Neuroinflammatory Markers

Neuroinflammatory biomarkers were quantified utilizing rat-specific ELISA kits. All assays were conducted employing a double-antibody sandwich protocol to ensure high specificity and sensitivity towards the target proteins. The COX-2 ELISA kit (Catalog No. 266603) was employed to assess endogenous COX-2 levels within brain homogenates. In this procedure, monoclonal anti-rat COX-2 antibodies are pre-coated onto microplate wells, followed by incubation with samples and biotin-labeled detection antibodies. Signal amplification is achieved using a streptavidin–HRP conjugate, and absorbance is measured at 450 nm to determine COX-2 concentrations. Similarly, TNF-α (Catalog No. 162068) and IL-6 (Catalog No. 269892) levels were quantified utilizing optimized sandwich ELISA kits specifically designed for rat cytokine detection. These assays employ capture and detection antibodies specific to each cytokine, enabling precise measurement of their levels in brain tissue samples.

#### 2.7.3. Bioassay of Apoptotic Proteins

The evaluation of apoptosis-related proteins was conducted utilizing rat-specific ELISA immunoassay kits. Each kit employs a double-antibody sandwich ELISA methodology to ensure high specificity and sensitive detection of target proteins within brain tissue homogenates. The BCL-2 ELISA Kit (Catalog No. 452319), BAX ELISA Kit (Catalog No. 2703209), and Caspase-3 ELISA Kit (Catalog No. 261814) incorporate monoclonal capture antibodies coated on microplate wells, followed by incubation with biotinylated detection antibodies and a streptavidin–HRP conjugate, enabling colorimetric measurement at 450 nm. These assays enable precise quantification of anti-apoptotic and pro-apoptotic protein levels across experimental groups.

#### 2.7.4. Bioassay Oxidative Markers

Oxidative stress-related biomarkers were measured using rat-specific ELISA kits. ELISA-based assays were employed because they provide sensitive, specific, and reproducible quantification of oxidative stress biomarkers. Compared with conventional biochemical methods, ELISA offers improved analytical specificity through antibody-based detection and allows methodological consistency with the assessment of inflammatory and apoptotic markers performed in the present study. The MDA ELISA kit (Catalog No. 738685) was utilized to evaluate oxidative stress in brain homogenates. According to the manufacturer’s specifications, the MDA ELISA kit demonstrated a detection range of 50–1000 ng/mL, a sensitivity of 1.0 ng/mL, an intra-assay coefficient of variation (CV) of <10%, and an inter-assay CV of <12%, with no significant cross-reactivity reported for MDA analogs. The assay was based on a competitive ELISA format in which endogenous MDA in the samples competes with an MDA-HRP conjugate for binding to anti-MDA antibodies, and absorbance was measured at 450 nm to determine MDA concentrations.

Similarly, CAT (Catalog No. 2704433) and GSH (Catalog No. 775264) levels were quantified using rat-specific ELISA kits designed to evaluate antioxidant defense status in brain tissue homogenates. According to the manufacturers’ specifications, the CAT assay exhibited a detection range of 0.156–10 ng/mL, a sensitivity of <0.064 ng/mL, intra-assay and inter-assay coefficients of variation (CVs) of <10% and <12%, respectively, and no significant cross-reactivity with related analogs. The GSH assay was based on biotin double-antibody sandwich ELISA technology, with a detection range of 0.1–8 ng/mL, a sensitivity of 0.01 ng/mL, an intra-assay CV of <10%, and an inter-assay CV of <15%. Both assays employed highly specific capture and detection antibodies for the quantitative determination of CAT and GSH levels in brain tissue homogenates.

### 2.8. Statistical Analysis

Data were analyzed by calculating mean values along with measures of variability (SEM). Longitudinal data, including body weight and fasting blood glucose levels, were analyzed using two-way repeated-measures ANOVA, with treatment group as the between-subject factor and time (weeks) as the within-subject factor. Bonferroni-adjusted multiple comparisons were performed to assess differences between treatment groups at individual time points. Other experimental outcomes (neurobehavioral assessments and biomolecular evaluations) involving comparisons among multiple independent groups were analyzed using one-way ANOVA followed by Tukey–Kramer multiple comparison testing. Statistical analyses were performed using GraphPad Prism (version 5.0; GraphPad Software, San Diego, CA, USA). The sample size (*n* = 6/group) was determined a priori based on power analysis and previous experimental evidence. Exact *p*-values from the ANOVA analyses are reported where available, and statistical significance was considered at *p* ≤ 0.05.

## 3. Results

### 3.1. Marked Modulation of Fasting Body Weight by Diabetes-Induced Animals (DIA) and SMG

**Figure 3:**
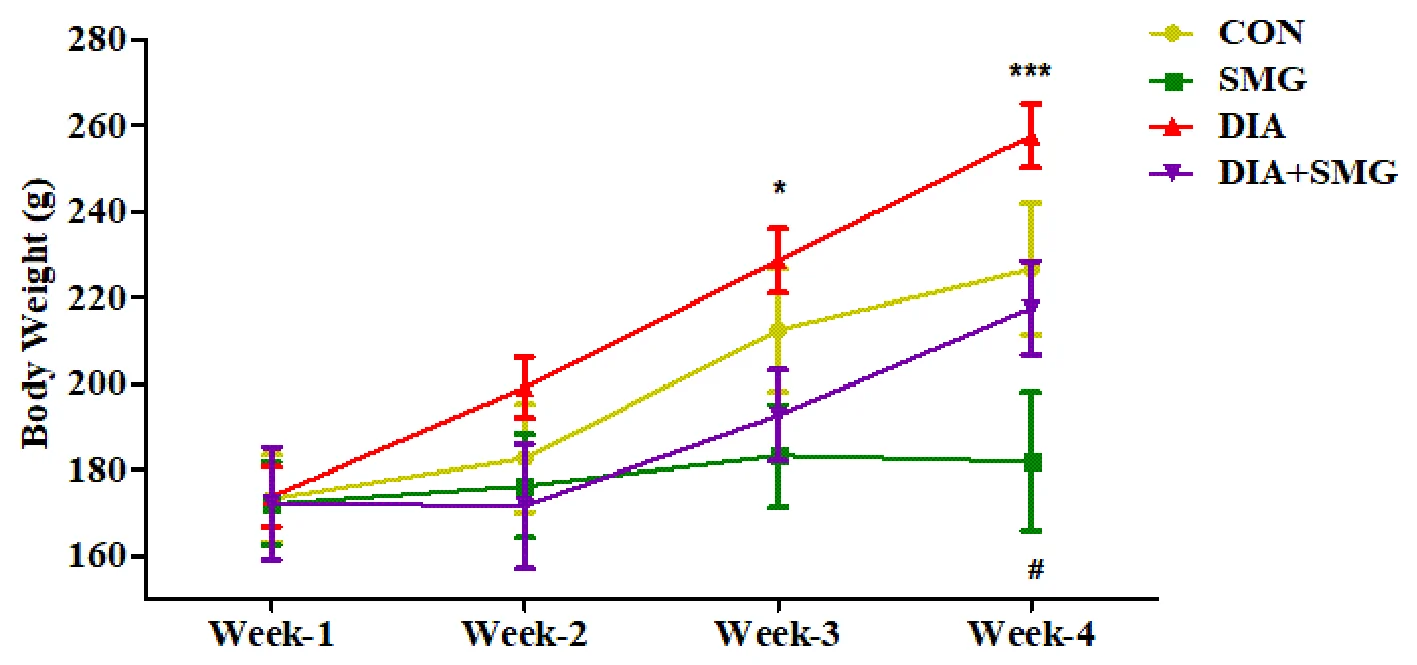
Changes in fasting body weight were recorded over the 30-day experimental period in all treatment groups. Data are expressed as mean ± SEM (*n* = 6/group). Statistical analysis was performed using two-way repeated-measures ANOVA followed by Bonferroni-adjusted multiple comparisons between treatment groups at individual time points. Statistical significance is denoted as # *p* < 0.05 versus CON; * *p* < 0.05 and *** *p* < 0.001 compared with the SMG group.

Body weight was analyzed using two-way repeated-measures ANOVA, with treatment group as the between-subject factor and time as the within-subject factor (Figure 3). The analysis revealed a significant main effect of time (weeks) [F(3,60) = 39.76, *p* < 0.0001] and a significant interaction between treatment group and weeks [F(9,60) = 3.98, *p* = 0.0005], whereas the main effect of treatment group was not statistically significant [F(3,20) = 2.43, *p* = 0.0948]. Bonferroni-adjusted multiple comparisons between treatment groups at individual time points revealed no significant differences during Weeks 1 and 2. The DIA group showed significantly higher body weight than the SMG group at Week 3 (*p* < 0.05) and Week 4 (*p* < 0.001). The CON group also showed significantly higher body weight than the SMG group at Week 4 (*p* < 0.05). No other between-group comparisons reached statistical significance.

### 3.2. Marked Modulation of Fasting Blood Glucose Levels by DIA and SMG

**Figure 4:**
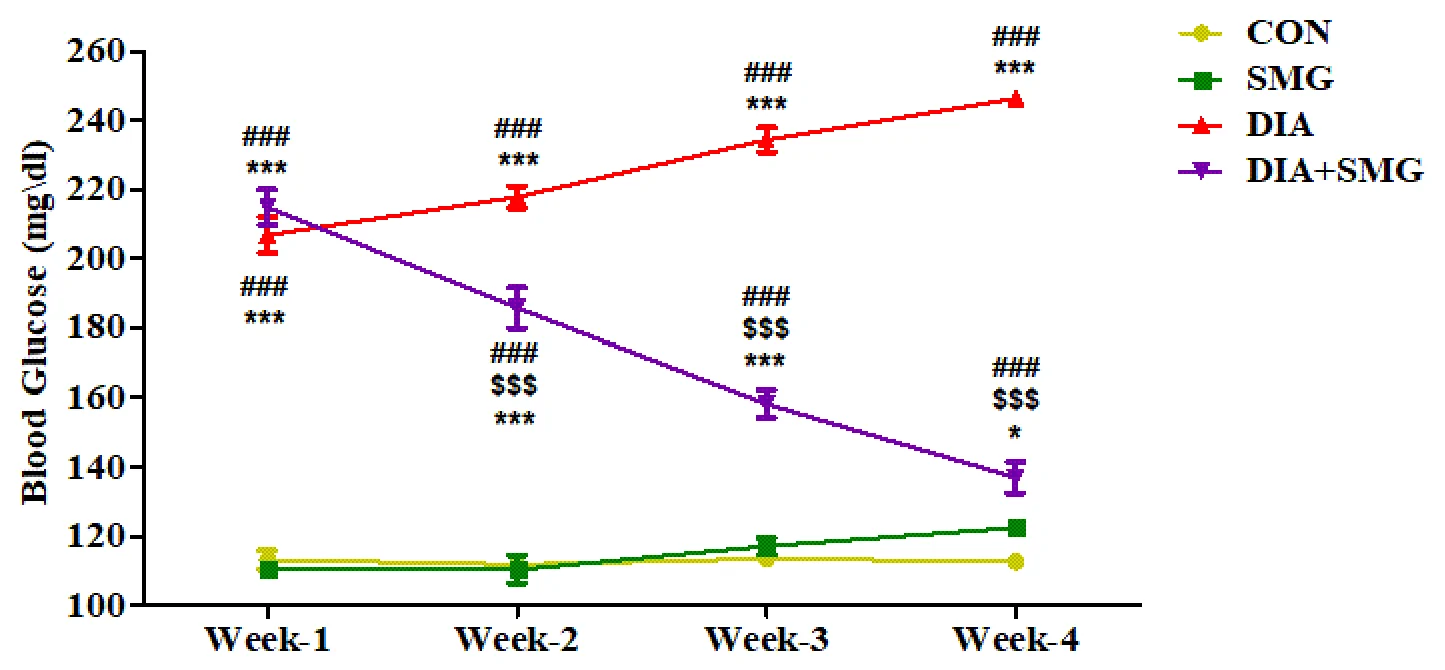
Fasting blood glucose levels were monitored across the 30-day treatment period in all experimental groups. Data are presented as mean ± SEM (*n* = 6/group). Statistical analysis was performed using two-way repeated-measures ANOVA followed by Bonferroni-adjusted multiple comparisons between treatment groups at individual time points. Significance markers: ### *p* < 0.001 versus CON; * *p* < 0.05 and *** *p* < 0.001 versus SMG; $$$ *p* < 0.001 versus the DIA group.

Figure 4 highlights pronounced changes in fasting blood glucose levels across the experimental period. Two-way repeated-measures ANOVA revealed significant effects of treatment group [F(3,20) = 600.6, *p* < 0.0001] and time (weeks) [F(3,60) = 3.645, *p* = 0.0175], together with a significant interaction between treatment group and weeks [F(9,60) = 49.72, *p* < 0.0001]. Bonferroni-adjusted multiple comparisons showed that the CON and SMG groups maintained blood glucose levels below 125 mg/dL throughout the experimental period, whereas the DIA and DIA+SMG groups exhibited blood glucose levels above 200 mg/dL at Week 1. The DIA group showed a progressive increase in blood glucose levels over time, reaching 246.3 mg/dL at Week 4, whereas the DIA+SMG group demonstrated a marked decline from 214.8 mg/dL at Week 1 to 137.0 mg/dL at Week 4. Blood glucose levels were significantly higher in the DIA group than in the CON and SMG groups at all four time points (*p* < 0.001). Importantly, the DIA+SMG group showed significantly lower blood glucose levels than the DIA group from Week 2 onward (*p* < 0.001), while no significant difference was observed between these groups at Week 1.

### 3.3. Marked Modulation of Transfer Latency (TL) Duration in EPM by DIA and SMG

**Figure 5:**
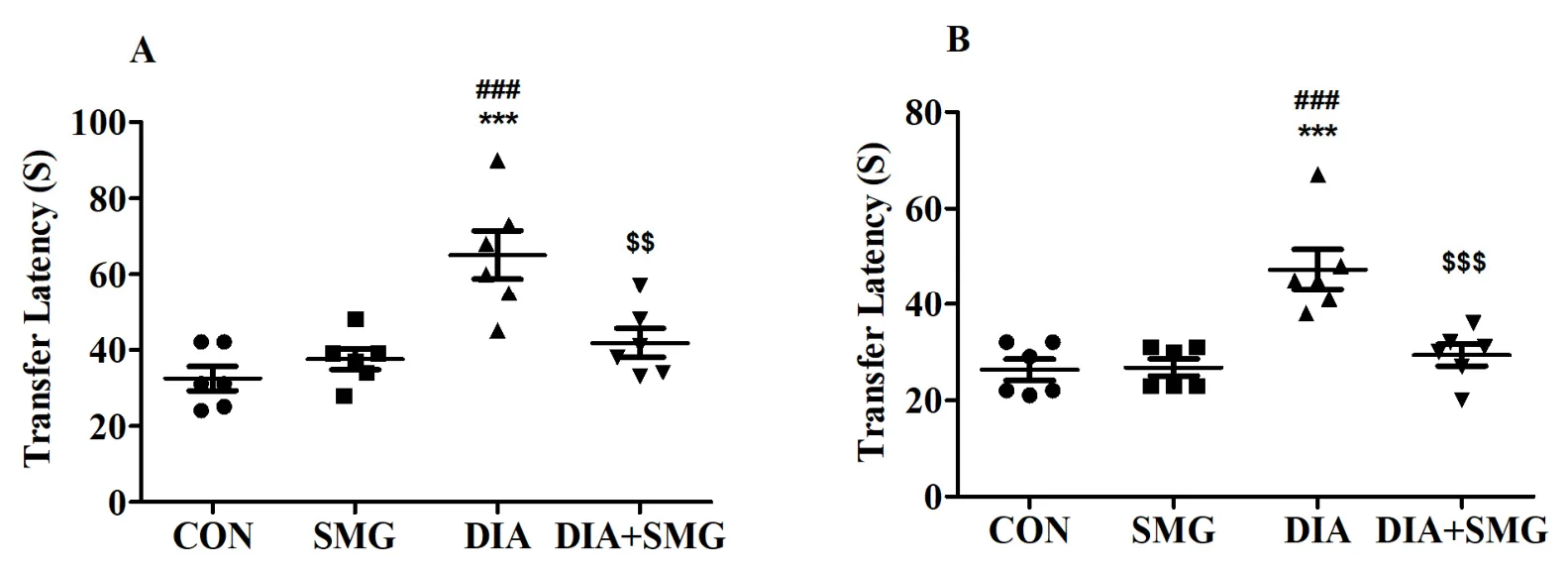
Effects of SMG and DIA treatments on cognitive performance in the EPM task, assessed through TL. Panel (**A**) shows TL values recorded on Day 1, and Panel (**B**) displays TL values on Day 2. Data are expressed as mean ± SEM (*n* = 6/group). Group differences were evaluated using one-way ANOVA [Day 1: F(3,20) = 11.52, *p* = 0.0001; Day 2: F(3,20) = 13.36, *p* < 0.0001], followed by Tukey–Kramer post hoc comparisons. Statistical significance is denoted as ### *p* < 0.001 versus CON; *** *p* < 0.001 versus SMG; $$ *p* < 0.01 and $$$ *p* < 0.001 versus the DIA group.

One-way ANOVA clearly demonstrated pronounced and statistically significant differences in TL among groups on both days, Day 27 [F(3,20) = 11.52, *p* = 0.0001] and Day 28 [F(3,20) = 13.36, *p* < 0.0001] (Figure 5). To examine these group variations in more detail, a Tukey–Kramer post hoc comparison was performed. Figure 5A illustrates the initial phase of the EPM test; the DIA group exhibited a significantly higher duration compared to both the CON and SMG groups, with a *p*-value less than 0.001. Notably, the DIA + SMG group demonstrated a reduced duration relative to the DIA group, with a *p*-value less than 0.01. Furthermore, comparison between the CON and SMG groups revealed no significant difference.

In the second phase, a consistent pattern emerged across all experimental groups, with only slight differences in *p*-value significance. Figure 5B shows the second part of the EPM test, where the DIA group spent significantly more time than both the CON and SMG groups, with a *p*-value under 0.001. Importantly, the DIA + SMG group had a shorter duration compared to the DIA group, also with a *p*-value below 0.001. Additionally, no meaningful difference was detected between the CON and SMG groups, indicating a significant reduction in transfer latency in the DIA + SMG group compared with the untreated diabetic group.

### 3.4. Marked Modulation of Neurobehavioral Performance in the NOR Test by DIA and SMG

**Figure 6:**
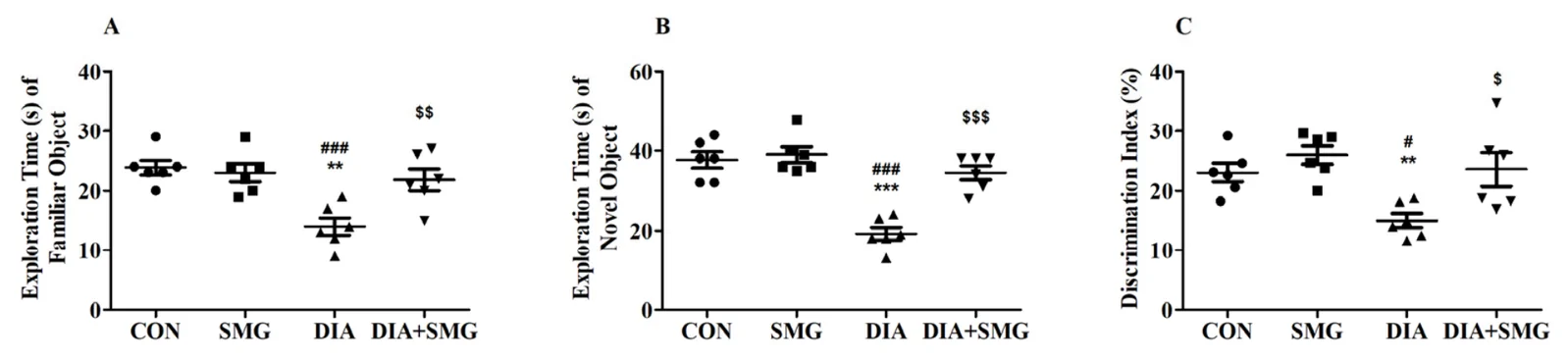
Effects of SMG and DIA treatments on cognitive performance in the Novel Object Recognition (NOR) task, evaluated through exploratory behavior and discrimination indices. Panel (**A**) shows the exploration time of the familiar object (EFO), Panel (**B**) depicts the exploration time of the novel object (ENO), and Panel (**C**) presents the percent discrimination index (PDI). Data are expressed as mean ± SEM (*n* = 6/group). Group comparisons were performed using one-way ANOVA [EFO: F(3,20) = 9.226, *p* = 0.0005; ENO: F(3,20) = 24.48, *p* < 0.0001; PDI: F(3,20) = 6.471, *p* = 0.0031], followed by Tukey–Kramer multiple comparison tests. Statistical significance: # *p* < 0.05 and ### *p* < 0.001 versus CON; ** *p* < 0.01 and *** *p* < 0.001 versus SMG; $ *p* < 0.05, $$ *p* < 0.01, and $$$ *p* < 0.001 versus the DIA group.

Figure 6A demonstrated [F(3,20) = 9.226, *p* = 0.0005], indicating a significant variation among all experimental groups in the Exploration time of familiar object (EFO) results of the one-way analysis of ANOVA. To examine these differences in more detail, a Tukey–Kramer post hoc evaluation was conducted. The EFO parameter was measured in seconds. The DIA group demonstrated a markedly shorter duration in comparison to both the CON and SMG groups, with *p*-values less than 0.001 and 0.01, in the same sequence. Notably, the DIA + SMG group exhibited an increased duration relative to the DIA group, with a *p*-value of less than 0.01. Additionally, no statistically significant differences were identified between other groups.

In Figure 6B, the Exploration time of the novel object (ENO) evaluation outcomes also showed significant differences among all groups [Day 2: ENO: F(3,20) = 24.48, *p* < 0.0001]. Post *hoc* tests showed that all experimental groups experienced notable reductions in time compared to the CON group. In the second phase, a consistent pattern emerged across all experimental groups, with only minor differences in *p*-values. The DIA group spent significantly less time than both the CON and SMG groups, with a *p*-value of less than 0.001. Notably, the DIA + SMG group demonstrated a longer duration than the DIA group, with a *p*-value below 0.001. Furthermore, no statistically significant differences were identified between other groups.

Figure 6C illustrates the PDI results, in which ANOVA analysis demonstrated notable changes across all groups [F(3,20) = 6.471, *p* = 0.0031]. To examine these Statistical variations in more detail, a Tukey–Kramer post hoc evaluation was conducted. The DIA group spent substantially less time than both the CON and SMG groups, with *p*-values of 0.05 and 0.01, in the same sequence. Notably, the DIA + SMG group had a longer duration than the DIA group, with a *p*-value less than 0.05. Furthermore, no statistically significant difference was observed between the CON and SMG groups.

### 3.5. Marked Modulation of Key Neuroinflammatory Markers in the Rat Brain by DIA and SMG

**Figure 7:**
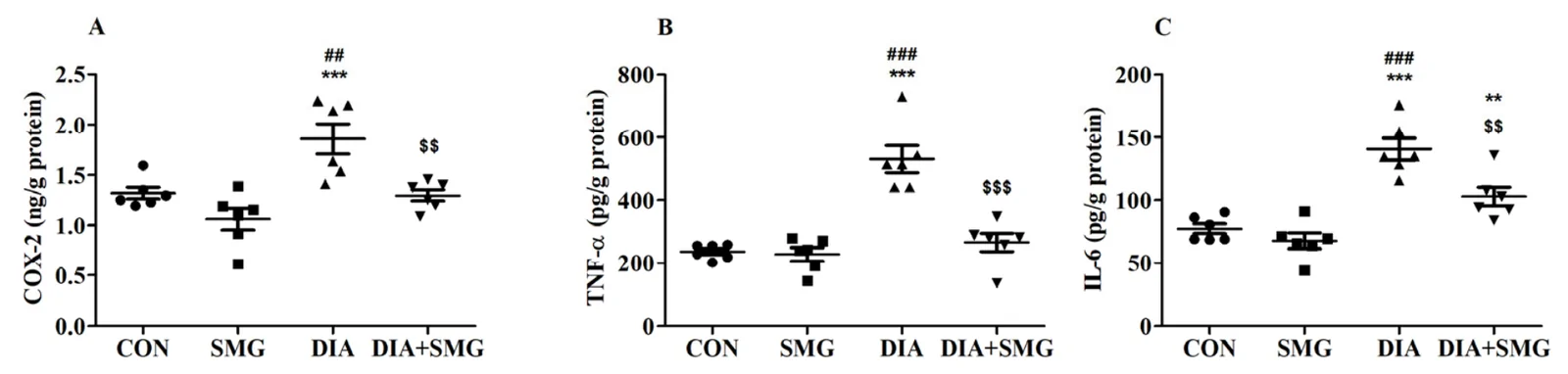
Effects of SMG and DIA treatments on key inflammatory markers in rat brain tissue. Panel (**A**) illustrates COX-2 levels, Panel (**B**) shows TNF-α concentrations, and Panel (**C**) presents IL-6 levels. Data are reported as mean ± SEM (*n* = 6/group). Statistical analysis was conducted using one-way ANOVA [COX-2: F(3,20) = 11.06, *p* = 0.0002; TNF-α: F(3,20) = 25.85, *p* < 0.0001; IL-6: F(3,20) = 23.03, *p* < 0.0001], followed by Tukey–Kramer post hoc comparisons. Significance indicators: ## *p* < 0.01 and ### *p* < 0.001 versus CON; ** *p* < 0.01 and ***p < 0.001 versus SMG; $$ *p* < 0.01 and $$$ *p* < 0.001 versus the DIA group.

The examination of neuroinflammatory biomarkers, including COX-2, TNF-α, and IL-6 levels across experimental groups, demonstrated notable variations, as determined by one-way ANOVA [COX-2: F(3,20) = 11.06, *p* = 0.0002; TNF-α: F(3,20) = 25.85, *p* < 0.0001; IL-6: F(3,20) = 23.03, *p* < 0.0001]. Subsequently, the Tukey–Kramer post hoc test was employed, as demonstrated in Figure 7. The DIA group exhibited the highest concentration of COX-2 among all groups. Specifically, the DIA group illustrated significantly higher levels than both the CON and SMG groups, with *p*-values of less than 0.01 and 0.001, in the same sequence. Notably, the DIA + SMG group had a lower concentration compared to the DIA group, with a *p*-value below 0.01. Furthermore, no statistically significant differences were identified between other groups, as shown in Figure 7A.

In Figure 7B, TNF-α levels in the DIA group were significantly higher than those in both the CON and SMG groups, with *p*-values less than 0.001. Notably, the DIA + SMG group exhibited a lower concentration compared to the DIA group, with a *p*-value below 0.001. No statistically significant differences were identified between other groups.

IL-6 results exhibit a pattern similar to those of COX-2 and TNF-α results (Figure 7C). IL-6 levels in the DIA group were markedly elevated relative to both the CON and SMG groups, with *p*-values less than 0.001. Notably, the DIA + SMG group demonstrated a reduced concentration in comparison to the DIA and CON groups, with a *p*-value below 0.01. Additionally, no statistically significant differences were identified between other groups.

### 3.6. Marked Modulation of Key Neuroapoptotic Markers in the Rat Brain by DIA and SMG

**Figure 8:**
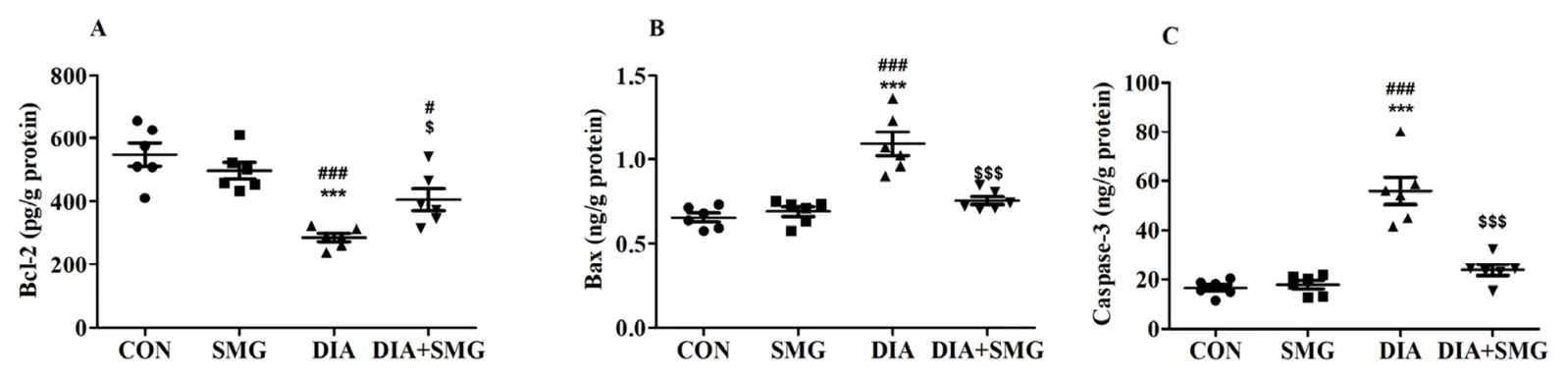
Effects of SMG and DIA treatments on apoptotic markers in rat brain tissue. Panel (**A**) shows Bcl-2 levels, Panel (**B**) displays Bax concentrations, and Panel (**C**) presents Caspase-3 levels. Data are expressed as mean ± SEM (*n* = 6/group). Statistical evaluation was performed using one-way ANOVA [Bcl-2: F(3,20) = 15.58, *p* < 0.0001; Bax: F(3,20) = 22.68, *p* < 0.0001; Caspase-3: F(3,20) = 34.52, *p* < 0.0001], followed by Tukey–Kramer multiple comparison testing. Significance indicators: # *p* < 0.05 and ### *p* < 0.001 versus CON; *** *p* < 0.001 versus SMG; $ *p* < 0.05 and $$$ *p* < 0.001 versus the DIA group.

The examination of neuroapoptosis biomarkers, including Bcl-2, Bax, and Caspase-3 levels across experimental groups, demonstrated notable variations, as determined by one-way ANOVA [Bcl-2: F(3,20) = 15.58, *p* < 0.0001; Bax: F(3,20) = 22.68, *p* < 0.0001; Caspase-3: F(3,20) = 34.52, *p* < 0.0001]. Following that, the Tukey–Kramer post hoc test was employed, as illustrated in Figure 8. Bcl-2 levels exhibited no significant difference between the CON and SMG groups; however, both groups demonstrated significantly increased levels compared to the DIA group, with *p*-values less than 0.001. Additionally, the DIA + SMG group illustrated a significant decrease in levels relative to the CON group, with *p*-values less than 0.05. The DIA + SMG group demonstrated a significantly attenuated effect compared with the DIA group alone, with *p*-values < 0.05, as shown in Figure 8A.

Figure 8B illustrates variations in Bax levels among the groups, with both the CON group and the SMG group exhibiting significantly lower levels compared to the DIA group, with *p*-values less than 0.001. Furthermore, SMG treatment was associated with significantly lower Bax levels compared with the untreated diabetic group, with *p*-values less than 0.001. No statistically significant differences were identified between other groups.

The Caspase-3 levels exhibited a pattern similar to that of Bax levels. The DIA group demonstrated a significant increase in Caspase-3 levels compared to all other experimental groups, with *p*-values less than 0.001. Furthermore, the DIA + SMG group showed no significant difference when compared to the CON group. No statistically significant differences were identified between other groups, as shown in Figure 8C.

### 3.7. Marked Modulation of Key Oxidative Stress Markers in the Rat Brain by DIA and SMG

**Figure 9:**
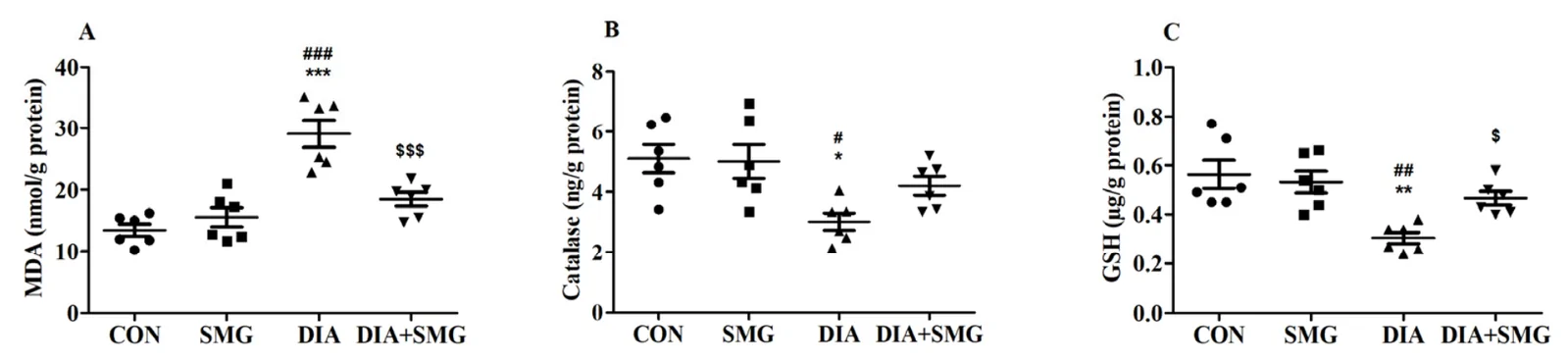
Effects of SMG and DIA treatments on oxidative markers in rat brain tissue. Panel (**A**) shows MDA concentrations, Panel (**B**) displays Catalase levels, and Panel (**C**) presents GSH concentrations. Data are expressed as mean ± SEM (*n* = 6/group). Statistical evaluation was performed using one-way ANOVA [MDA: F(3,20) = 20.10, *p* < 0.0001; Catalase: F(3,20) = 5.154, *p* = 0.0084; GSH: F(3,20) = 8.182, *p* = 0.0009], followed by Tukey–Kramer multiple comparison testing. Significance indicators: # *p* < 0.05, ## *p* < 0.01, and ### *p* < 0.001 versus CON; * *p* < 0.05, ** *p* < 0.01, and *** *p* < 0.001 versus SMG; $ *p* < 0.05 and $$$ *p* < 0.001 versus the DIA group.

Assessment of oxidative stress parameters, including MDA, catalase activity, and GSH levels, revealed notable changes across the experimental groups, as confirmed by one-way ANOVA [MDA: F(3,20) = 20.10, *p* < 0.0001; catalase: F(3,20) = 5.154, *p* = 0.0084; GSH: F(3,20) = 8.182, *p* = 0.0009], followed by Tukey–Kramer post hoc analysis, as depicted in Figure 9. In addition, as illustrated in Figure 9A, MDA levels were significantly higher in the DIA group than in both the CON and SMG groups (*p* < 0.001), indicating increased lipid peroxidation and oxidative damage. Treatment with SMG significantly reduced MDA levels in the DIA + SMG group, in contrast to the DIA group alone (*p* < 0.001), indicating reduced MDA levels in the SMG-treated diabetic group compared with the untreated diabetic group. No statistically significant differences were identified between the CON and SMG groups.

Figure 9B shows that catalase activity did not differ significantly between the CON and SMG groups. In contrast, the DIA group exhibited a significant reduction in catalase activity compared with both groups (*p* < 0.05), consistent with reduced antioxidant capacity. Administration of SMG in the DIA + SMG group significantly increased catalase activity compared with the DIA group (*p* < 0.05), indicating partial restoration of enzymatic antioxidant capacity, although values remained below those of the control groups. No statistically significant differences were identified between other groups.

Similarly, as illustrated in Figure 9C, GSH concentrations were remarkably lower in the DIA group than in the CON and SMG groups (*p* < 0.01), indicating depletion of endogenous antioxidant reserves. Notably, the DIA + SMG group showed a significant increase in GSH levels relative to the DIA group alone (*p* < 0.05), suggesting partial improvement in antioxidant status. No statistically significant differences were identified between the CON and SMG groups.

## 4. Discussion

The present study demonstrated that T2DM induction was associated with behavioral impairments, increased neuroinflammatory and apoptotic markers, and disruption of oxidative stress parameters in rat brain tissue. These findings are consistent with previous evidence indicating that metabolic dysregulation and chronic hyperglycemia contribute to cognitive dysfunction and neuronal injury in diabetes [8,35]. Oxidative stress has been proposed as one of the factors involved in the progression of diabetic complications, including those affecting the CNS [36]. However, previous studies have largely evaluated cognitive effects using injectable semaglutide or investigated oral administration in non-cognitive or peripheral neurological models. In contrast, the present study evaluated the clinically available oral formulation of semaglutide (Rybelsus^®^) in a nicotinamide/streptozotocin-induced T2DM model, combining two behavioral paradigms (EPM and NOR) with an integrated assessment of neuroinflammatory, oxidative stress, and apoptosis-related biomarkers in brain tissue. Thus, the present findings extend the existing evidence by characterizing the association between oral semaglutide treatment and diabetes-associated neurobehavioral and brain biochemical alterations under these experimental conditions. However, these findings should not be interpreted as evidence of a direct neuroprotective mechanism, as the observed improvements may have been influenced, at least in part, by the improvement in glycemic and metabolic control following SMG treatment. In the current study, SMG treatment was associated with attenuation of several diabetes-related alterations, suggesting a potential beneficial effect of SMG on diabetes-associated neurobehavioral and biochemical alterations under these experimental conditions. These observations are consistent with previous reports describing beneficial effects of GLP-1 receptor agonists in experimental models of metabolic and neurological dysfunction [37]. The present study evaluated the clinically available oral formulation of semaglutide (Rybelsus^®^), providing preclinical evidence of an association between oral SMG treatment and improved diabetes-associated neurobehavioral and biochemical alterations under these experimental conditions. Given the increasing clinical use of oral SMG in T2DM management, these findings enhance the translational relevance of the study.

However, the observed improvements in neurobehavioral performance and biochemical parameters may not be attributed exclusively to direct central effects of SMG. The improvement in glycemic control following SMG treatment may have contributed, at least partially, to the attenuation of oxidative stress, inflammation, and apoptosis-related alterations observed in the diabetic brain. Since the present study was not designed to investigate specific molecular mechanisms or distinguish direct neuronal actions from secondary metabolic effects, the findings should be interpreted as an association between oral SMG treatment and improved diabetes-associated alterations. Further mechanistic studies are required to clarify the precise pathways involved.

SMG treatment was associated with lower body weight compared with untreated diabetic animals, consistent with its established metabolic effects and previous reports [38]. Because improved metabolic control may itself contribute to improved neurological outcomes, these findings further support the need for future mechanistic studies capable of distinguishing direct neuroprotective effects from secondary metabolic effects.

The EPM is a commonly used behavioral test to assess learning and memory, based on rodents’ natural avoidance of open, elevated spaces in favor of enclosed areas, with shorter transfer latency indicating better spatial memory [39]. The NOR test measures rats’ ability to identify, engage with, and differentiate between a familiar object and a new one over time. This test is grounded in the natural behavior of rats, which show a stronger inclination to explore unfamiliar objects they have not encountered before [40]. Individuals with T2DM encounter distinct difficulties in initiating and sustaining healthy lifestyle behaviors, partly due to changes in brain function and neural plasticity that can cause cognitive decline [41]. The EPM test showed the DIA group had significant neurobehavioral impairments, indicated by increased TL in both phases. These suggest deficits in spatial learning and memory, likely due to DIA-related metabolic stress causing hippocampal dysfunction and disrupted synaptic function and plasticity [42,43]. Interestingly, the DIA + SMG group showed a significant reduction in neurobehavioral toxicity compared to the DIA group alone in both phases, indicating improved behavioral performance following SMG treatment. The reduction in transfer latency suggests improved spatial memory performance following SMG treatment under diabetic conditions. Conversely, a similar pattern was observed in the NOR test, including EFO, ENO, and C parameters. The DIA group exhibited a significant decrease across all parameters when compared to all other experimental groups. Cognitive deficits reflect dysfunction within hippocampal and prefrontal cortical regions, which are particularly vulnerable to oxidative and metabolic stress [44]. Furthermore, the combination group (DIA + SMG) demonstrated a significantly higher level compared to the DIA group, with no significant difference observed when compared to the CON group and the SMG alone group. This improvement suggests partial recovery of recognition memory and cognitive discrimination abilities following SMG treatment. The consistent improvement observed across both EPM and NOR paradigms supports the potential beneficial effects of SMG on diabetes-associated cognitive impairment. Overall, these findings suggest that oral SMG may exert beneficial effects on diabetes-associated cognitive impairment under the current experimental conditions. This aligns with a recently published paper indicating that the SMG drug suggested potential cognitive benefits [30].

Chronic low-grade inflammation has been implicated in the progression of both metabolic and neurodegenerative disorders, and T2DM is recognized as a risk factor for cognitive impairment and age-related neurological decline. Therefore, the inflammatory changes detected in the present study may reflect the contribution of diabetes-associated metabolic dysregulation to CNS dysfunction [45]. The elevated levels of IL-6, TNF-α, and COX-2 observed in the DIA group are consistent with activation of inflammatory pathways previously reported in diabetic and neurodegenerative conditions. These mediators have been widely associated with neuroinflammatory responses and may contribute to neuronal dysfunction under conditions of chronic metabolic stress. In the present study, SMG treatment was associated with lower concentrations of these inflammatory markers compared with the untreated diabetic group, suggesting a potential modulatory effect on diabetes-associated neuroinflammation [46,47]. Our findings demonstrated that the DIA group exhibited significantly elevated levels of COX-2, TNF-α, and IL-6 compared to all other experimental groups. Furthermore, the combination group (DIA + SMG) displayed a notably reduced level relative to the DIA group alone, which is consistent with an association between SMG treatment and reduced neuroinflammatory responses.

Apoptosis-related markers were assessed to examine neuronal responses associated with T2DM-induced neurotoxicity. The DIA group exhibited lower Bcl-2 levels and higher Bax and Caspase-3 levels compared with the other experimental groups, findings that are consistent with increased apoptotic activity in diabetic brain tissue. In contrast, the DIA + SMG group showed attenuation of these alterations, suggesting that SMG treatment may modulate apoptosis-related pathways under diabetic conditions. These findings are in agreement with previous studies linking oxidative stress and inflammation to apoptosis-associated neuronal injury [33,48,49]. However, the present results do not establish the specific molecular mechanisms involved and should be interpreted as evidence of an association between SMG treatment and changes in apoptosis-related biomarkers.

Oxidative stress markers were evaluated to assess redox alterations associated with T2DM-induced neurotoxicity. The DIA group exhibited increased MDA levels together with reduced catalase and GSH levels compared with the other experimental groups, findings that are consistent with increased oxidative stress and impaired antioxidant defense in diabetic brain tissue [50]. In contrast, the DIA + SMG group showed lower MDA levels and higher catalase and GSH levels relative to the untreated diabetic group. These changes suggest that SMG treatment was associated with partial improvement of oxidative stress-related alterations under diabetic conditions, indicating a potential effect on redox balance. Similar observations have been reported in previous studies investigating the relationship between diabetes, oxidative stress, and neuronal injury [51,52,53,54].

## 5. Study Limitations

The present study provides an integrated neurobehavioral and biochemical evaluation of oral SMG in a rat model of T2DM; however, several limitations should be acknowledged. Although representative biomarkers of neuroinflammation, oxidative stress, and apoptosis were assessed, the number of biomarkers included for each pathway was limited and may not fully reflect the complexity of the underlying biological processes. In addition, biochemical analyses were performed using whole brain homogenates, which do not allow assessment of region-specific alterations in vulnerable brain areas such as the hippocampus and cerebral cortex. The study included only male Sprague–Dawley rats; therefore, potential sex-related differences in the effects of SMG were not evaluated. Furthermore, no histopathological or immunohistochemical assessments were performed to further validate the biochemical findings at the tissue level. The treatment duration was limited to 30 days, which may not fully represent the long-term neurological consequences of T2DM or the sustained effects of oral SMG administration. An important limitation of the present study is that no mechanistic experiments were performed to distinguish the direct neuroprotective effects of oral SMG from indirect effects secondary to improved glycemic control. Moreover, the present study was not designed to investigate the specific molecular mechanisms underlying the observed neurobehavioral and biochemical changes. Therefore, the contribution of glucose-independent effects to the observed neurobehavioral and biochemical improvements remains unclear. Future studies should incorporate longer treatment durations, both sexes, region-specific analyses, mechanistic pathway investigations, and glucose-matched experimental designs to distinguish direct neuroprotective effects from indirect metabolic effects of oral SMG under diabetic conditions.

## 6. Conclusions

The present study demonstrated that T2DM induction was associated with metabolic disturbances, behavioral deficits, increased inflammatory and apoptosis-related biomarkers, and altered oxidative stress parameters in rat brain tissue. Oral semaglutide (Rybelsus^®^) treatment was associated with improvements in behavioral performance, fasting blood glucose, body weight, and several biochemical markers, including COX-2, TNF-α, IL-6, Bax, Caspase-3, MDA, GSH, catalase, and Bcl-2. These findings demonstrate an association between oral semaglutide treatment and attenuation of diabetes-associated neurobehavioral and biochemical alterations in this experimental model. However, the present findings do not establish whether these effects are mediated by direct central actions of semaglutide or secondary to improved metabolic control. Further studies are warranted to elucidate the underlying mechanisms, compare oral and injectable formulations, and determine the translational relevance of these findings in clinical settings.

## Data Availability

The data presented in this study are available in the article; further inquiries can be directed to the corresponding author.

## Abbreviations

The following abbreviations are used in this manuscript:

AD	Alzheimer’s disease
Bax	Bcl-2-associated X protein
BBB	Blood–brain barrier
Bcl-2	B-cell lymphoma 2
CAT	Catalase
CNS	Central nervous system
CON	Control group
COX-2	Cyclooxygenase-2
DALYs	Disability-adjusted life years
DIA	Diabetes-induced animals
DIA + SMG	Diabetes-induced animals treated with semaglutide
EFO	Exploration time of familiar object
ELISA	Enzyme-linked immunosorbent assay
ENO	Exploration time of novel object
EPM	Elevated plus maze
FBG	Fasting blood glucose
GLP-1	Glucagon-like peptide-1
GLP-1RA	Glucagon-like peptide-1 receptor agonist
GSH	Reduced glutathione
IL-1β	Interleukin-1 beta
IL-6	Interleukin-6
IL-8	Interleukin-8
MDA	Malondialdehyde
MCP-1	Monocyte chemoattractant protein-1
MMSE	Mini-Mental State Examination
NOR	Novel object recognition
NTH	Nicotinamide hydrochloride
PDI	Percent discrimination index
ROS	Reactive oxygen species
SEM	Standard error of the mean
SMG	Semaglutide
SNAC	Sodium N-(8-[2-hydroxybenzoyl]amino) caprylate
STZ	Streptozotocin
T2DM	Type 2 diabetes mellitus
TL	Transfer latency
TNF-α	Tumor necrosis factor-alpha

## References

[1] NCD Risk Factor Collaboration (NCD-RisC) (2024). Worldwide trends in diabetes prevalence and treatment from 1990 to 2022: A pooled analysis of 1108 population-representative studies with 141 million participants. Lancet.

[2] Młynarska E., Czarnik W., Dzieża N., Jędraszak W., Majchrowicz G., Prusinowski F., Stabrawa M., Rysz J., Franczyk B. (2025). Type 2 diabetes mellitus: New pathogenetic mechanisms, treatment and the most important complications. Int. J. Mol. Sci..

[3] Cadena Sandoval M., Haeusler R.A. (2025). Bile acid metabolism in type 2 diabetes mellitus. Nat. Rev. Endocrinol..

[4] Zhang H., Jia Q., Song P., Li Y., Jiang L., Fu X., Li S. (2025). Incidence, prevalence, and burden of type 2 diabetes in China: Trend and projection from 1990 to 2050. Chin. Med. J..

[5] Galicia-Garcia U., Benito-Vicente A., Jebari S., Larrea-Sebal A., Siddiqi H., Uribe K.B., Ostolaza H., Martín C. (2020). Pathophysiology of type 2 diabetes mellitus. Int. J. Mol. Sci..

[6] Hu F.B., Manson J.E., Stampfer M.J., Colditz G., Liu S., Solomon C.G., Willett W.C. (2001). Diet, lifestyle, and the risk of type 2 diabetes mellitus in women. N. Engl. J. Med..

[7] Schellenberg E.S., Dryden D.M., Vandermeer B., Ha C., Korownyk C. (2013). Lifestyle interventions for patients with and at risk for type 2 diabetes: A systematic review and meta-analysis. Ann. Intern. Med..

[8] Seaquist E.R. (2010). The final frontier: How does diabetes affect the brain?. Diabetes.

[9] Banks W.A. (2020). The blood–brain barrier interface in diabetes mellitus: Dysfunctions, mechanisms and approaches to treatment. Curr. Pharm. Des..

[10] Mani V., Arfeen M. (2024). In vivo and computational studies on sitagliptin’s neuroprotective role in type 2 diabetes mellitus: Implications for Alzheimer’s disease. Brain Sci..

[11] Johnson A.R., Milner J.J., Makowski L. (2012). The inflammation highway: Metabolism accelerates inflammatory traffic in obesity. Immunol. Rev..

[12] Kellogg A.P., Wiggin T.D., Larkin D.D., Hayes J.M., Stevens M.J., Pop-Busui R. (2007). Protective effects of cyclooxygenase-2 gene inactivation against peripheral nerve dysfunction and intraepidermal nerve fiber loss in experimental diabetes. Diabetes.

[13] Pop-Busui R., Marinescu V., Van Huysen C., Li F., Sullivan K., Greene D.A., Larkin D., Stevens M.J. (2002). Dissection of metabolic, vascular, and nerve conduction interrelationships in experimental diabetic neuropathy by cyclooxygenase inhibition and acetyl-L-carnitine administration. Diabetes.

[14] Abdulmalek S., Nasef M., Awad D., Balbaa M. (2021). Protective effect of natural antioxidant, curcumin nanoparticles, and zinc oxide nanoparticles against type 2 diabetes-promoted hippocampal neurotoxicity in rats. Pharmaceutics.

[15] Roberts R.O., Knopman D.S., Przybelski S.A., Mielke M.M., Kantarci K., Preboske G.M., Senjem M.L., Pankratz V.S., Geda Y.E., Boeve B.F. (2014). Association of type 2 diabetes with brain atrophy and cognitive impairment. Neurology.

[16] Yeung S.E., Fischer A.L., Dixon R.A. (2009). Exploring effects of type 2 diabetes on cognitive functioning in older adults. Neuropsychology.

[17] Yilgor A., Demir C. (2024). Determination of oxidative stress level and some antioxidant activities in refractory epilepsy patients. Sci. Rep..

[18] Hölscher C. (2014). Central effects of GLP-1: New opportunities for treatments of neurodegenerative diseases. J. Endocrinol..

[19] Zhu Y., He Y., Yang H., Gao Y., Wang Y., Liu P., Zhang M. (2025). Semaglutide ameliorates diabetes-associated cognitive dysfunction in mouse model of type 2 diabetes. PLoS ONE.

[20] Liu D.X., Zhao C.S., Wei X.N., Ma Y.P., Wu J.K. (2022). Semaglutide protects against 6-OHDA toxicity by enhancing autophagy and inhibiting oxidative stress. Park. Dis..

[21] Salem E.A., Alqahtani S.M., El-Shoura E.A.M., Zaghlool S.S., Abdelzaher L.A., Mohamed S.A.M., Alalhareth I.S., Sheref A.A.M. (2025). Neuroprotective effects of semaglutide and metformin against rotenone-induced neurobehavioral changes in male diabetic rats. Naunyn Schmiedebergs Arch. Pharmacol..

[22] Smits M.M., Van Raalte D.H. (2021). Safety of semaglutide. Front. Endocrinol..

[23] Chao A.M., Tronieri J.S., Amaro A., Wadden T.A. (2023). Semaglutide for the treatment of obesity. Trends Cardiovasc. Med..

[24] O’Neil P.M., Birkenfeld A.L., McGowan B., Mosenzon O., Pedersen S.D., Wharton S., Carson C.G., Jepsen C.H., Kabisch M., Wilding J.P.H. (2018). Efficacy and safety of semaglutide compared with liraglutide and placebo for weight loss in patients with obesity: A randomised, double-blind, placebo- and active-controlled, dose-ranging, phase 2 trial. Lancet.

[25] Marso S.P., Bain S.C., Consoli A., Eliaschewitz F.G., Jódar E., Leiter L.A., Lingvay I., Rosenstock J., Seufert J., Warren M.L. (2016). Semaglutide and cardiovascular outcomes in patients with type 2 diabetes. N. Engl. J. Med..

[26] Alawaji R., Abdel-Bakky M.S., Ali H.M., Aljuhani M.A., Alshammari A.A.A., Kamal H.K., Khoja M.M.K., Alsehemi K., Korani M.A., Said E.S. (2025). Assessing the therapeutic potential of tirzepatide in modulating inflammatory responses and mitigating acute pancreatitis. Anim. Model. Exp. Med..

[27] Nassar M., Nassar O., Abosheaishaa H., Misra A. (2024). Decreased risk of recurrent acute pancreatitis with semaglutide and tirzepatide in people with type 2 diabetes or obesity with a history of acute pancreatitis: A propensity-matched global federated TriNetX database-based retrospective cohort study. Diabetes Metab. Syndr..

[28] Fatima M.T., Bhat A.A., Nisar S., Fakhro K.A., Al-Shabeeb Akil A.S. (2023). The role of dietary antioxidants in type 2 diabetes and neurodegenerative disorders: An assessment of the benefit profile. Heliyon.

[29] Lee S.O., Kuthati Y., Huang W.H., Wong C.S. (2024). Semaglutide ameliorates diabetic neuropathic pain by inhibiting neuroinflammation in the spinal cord. Cells.

[30] Boboc I.K.S., Dumitrelea P.D., Meca A.D., Mititelu-Tartau L., Bogdan M. (2024). Exploring the impact of semaglutide on cognitive function and anxiety-related behaviors in a murine model of Alzheimer’s disease. Biomedicines.

[31] du Sert N.P., Hurst V., Ahluwalia A., Alam S., Avey M.T., Baker M., Browne W.J., Clark A., Cuthill I.C., Dirnagl U. (2020). The ARRIVE guidelines 2.0: Updated guidelines for reporting animal research. PLoS Biol..

[32] Sharma A.C., Kulkarni S.K. (1992). Evaluation of learning and memory mechanisms employing elevated plus-maze in rats and mice. Prog. Neuro-Psychopharmacol. Biol. Psychiatry.

[33] Alshammari A.A.A., Arfeen M., Alkhamiss A.S., Alwesmi M.B., Mani V. (2025). Montelukast’s potential as a neuroprotective agent against acrylamide-induced neurotoxicity: In vivo and computational modelling. Food Chem. Toxicol..

[34] Lueptow L.M. (2017). Novel object recognition test for the investigation of learning and memory in mice. J. Vis. Exp..

[35] Liu Q., Dai S., Li X., Chen Y., Wang N., Wu J., Zhou Y., Yan B., Guo Y., Liu Y. (2025). A review of brain research on T2DM-related cognitive dysfunction. Open Med. (Wars.).

[36] Shrivastav D., Dabla P.K., Sharma J., Viswas A., Mir R. (2023). Insights on antioxidant therapeutic strategies in type 2 diabetes mellitus: A narrative review of randomized control trials. World J. Diabetes.

[37] Marassi M., Fadini G.P. (2025). Real-world evidence on oral semaglutide for the management of type 2 diabetes: A narrative review for clinical practice. Clin. Ther..

[38] Rodriguez P.J., Goodwin Cartwright B.M., Gratzl S., Brar R., Baker C., Gluckman T.J., Stucky N.L. (2024). Semaglutide vs tirzepatide for weight loss in adults with overweight or obesity. JAMA Intern. Med..

[39] Alshammari A.A.A., Almutairi A.B., Arfeen M., Alkhamiss A.S., Aldubayan M.A., Alhowail A.H., Mani V. (2024). Assessing the influence of intermittent alcohol access on acrylamide-induced neuronal toxicity in an experimental rat model. Brain Sci..

[40] Coirini H., Rey M., Gonzalez Deniselle M.C., Kruse M.S. (2022). Long-term memory function impairments following sucrose exposure in juvenile versus adult rats. Biomedicines.

[41] Cabral D.F., Bigliassi M., Morris T.P., Gomes-Osman J.R., Fried P.J. (2025). Integrating neural substrates, diabetes self-management, and behavior change for tailored lifestyle interventions in type 2 diabetes: A neurobehavioral perspective. Neurosci. Biobehav. Rev..

[42] Kim E.J., Pellman B., Kim J.J. (2015). Stress effects on the hippocampus: A critical review. Learn. Mem..

[43] Liu L., Zhou X., Zhang Y., Pu J., Yang L., Yuan S., Zhao L., Zhou C., Zhang H., Xie P. (2018). Hippocampal metabolic differences implicate distinctions between physical and psychological stress in four rat models of depression. Transl. Psychiatry.

[44] Zahedi E., Kavyannejad R., Salami P., Sadr S.S. (2025). Modulating depressive-like behaviors, memory impairment, and oxidative stress in chronic stress rat model using visible light therapy. Sci. Rep..

[45] Kacem H., d’Angelo M., Qosja E., Topi S., Castelli V., Cimini A. (2025). The inflammatory bridge between type 2 diabetes and neurodegeneration: A molecular perspective. Int. J. Mol. Sci..

[46] Rauf A., Badoni H., Abu-Izneid T., Olatunde A., Rahman M.M., Painuli S., Semwal P., Wilairatana P., Mubarak M.S. (2022). Neuroinflammatory markers: Key indicators in the pathology of neurodegenerative diseases. Molecules.

[47] Shastri A., Bonifati D.M., Kishore U. (2013). Innate immunity and neuroinflammation. Mediat. Inflamm..

[48] Kilanowska A., Ziółkowska A. (2022). Apoptosis in type 2 diabetes: Can it be prevented? Hippo pathway prospects. Int. J. Mol. Sci..

[49] Radi E., Formichi P., Battisti C., Federico A. (2014). Apoptosis and oxidative stress in neurodegenerative diseases. J. Alzheimers Dis..

[50] Dash U.C., Bhol N.K., Swain S.K., Samal R.R., Nayak P.K., Raina V., Panda S.K., Kerry R.G., Duttaroy A.K., Jena A.B. (2025). Oxidative stress and inflammation in the pathogenesis of neurological disorders: Mechanisms and implications. Acta Pharm. Sin. B.

[51] Alhumaydhi F.A., Younus H., Khan M.A. (2025). Catalase functions and glycation: Their central roles in oxidative stress, metabolic disorders, and neurodegeneration. Catalysts.

[52] Chandimali N., Bak S.G., Park E.H., Lim H.J., Won Y.S., Kim E.K., Park S.I., Lee S.J. (2025). Free radicals and their impact on health and antioxidant defenses: A review. Cell Death Discov..

[53] Georgiou-Siafis S.K., Tsiftsoglou A.S. (2023). The key role of GSH in keeping the redox balance in mammalian cells: Mechanisms and significance of GSH in detoxification via formation of conjugates. Antioxidants.

[54] Rasheed Z. (2024). Therapeutic potentials of catalase: Mechanisms, applications, and future perspectives. Int. J. Health Sci..

